# COVID-19 vs. Cancer Immunosurveillance: A Game of Thrones within an Inflamed Microenviroment

**DOI:** 10.3390/cancers14174330

**Published:** 2022-09-05

**Authors:** Ioannis Liapis, Stavroula Baritaki

**Affiliations:** Laboratory of Experimental Oncology, Division of Surgery, School of Medicine, University of Crete, GR-71003 Heraklion, Greece

**Keywords:** cancer, tumor immunosurveillance, COVID-19, infection

## Abstract

**Simple Summary:**

Cancer immunosurveillance exists as a mechanism of efficient eradication of tumor establishment and progression. On the flip side, chronic inflammation functions as an ultimate background that may favor cancer development and advancement. Severe infection by the recently emerged SARS-CoV-2 virus often causes a multifaceted inflammation with acute and chronic characteristics that may affect several organs. The current review discusses the hypothesis of a potential interplay between the mechanisms of cancer immunosurveillance and the COVID-19-sustained inflammation, as well as the putative consequences that this cross-talk may have in oncogenesis and tumor progression.

**Abstract:**

The COVID-19 pandemic accounts for more than 500 million confirmed infections and over 6 million deaths worldwide in the last 2 years. SARS-CoV-2 causes a highly complex form of inflammation that affects the human organism both acutely and chronically. In the same line, cancer as an inflammation-induced and immune-editing disease appears to cross-react with immune system at different levels including early interactions during carcinogenesis and later cross-talks within the tumor microenvironment. With all that in mind, a reasonable question one might address is whether the SARS-CoV-2 infection and the derived “long lasting inflammatory status” that is frequently observed in patients, might affect the cancer immunosurveillance mechanisms and consequently their risk of developing cancer, as well as the tumor and immune cell behaviors within the inflamed microenvironment. On this context, this review intends to outline and discuss the existing knowledge on SARS-CoV-2-mediated immunomodulation under the prism of changes that might be able to interfere with cancer cell immunoescape and the overall tumor progression and response to conventional therapeutics. Our goal is to highlight a potential interplay between the COVID-19 immunopathology and cancer immune-microenvironment that may pave the way for thorough investigation in the future.

## 1. Introduction

As of June 2022, the World Health Organization (WHO) had reported over five hundred million confirmed cases of infections with the Severe Acute Respiratory Syndrome Coronavirus 2 (SARS-CoV-2) and over six million associated deaths, globally [1]. The main load of the reported cases mainly concerned the developed American and European countries, where the confirmed numbers have exceeded the 160 and 230 million, respectively [1]. Although infection rates have not shown any significant association with gender and age, the severity of the disease and the final outcome seem to worsen as the age of infected patients increases [2]. Following the primary infections by the initial SARS-CoV-2 strain, many variants of the virus have emerged thereafter, especially after the onset of the vaccination programs globally [1], thus resulting in a significant increase of the disease-associated cases and deaths. Notably, the USA alone counts over a million deaths, despite that more than 500 million vaccines have already been administered.

Recent advances in our understanding of SARS-CoV-2 structure and pathophysiology have revealed that the virus contains four important structural proteins: the spike (S), the membrane (M), the envelope (E) and the nucleocapsid (N) proteins, while it is transmitted via respiratory droplets [3]. The virus binds the ciliated cells in the nasal mucosa, via the interaction of the S protein with the angiotensin converting enzyme II (ACE II) [4]. This leads to S protein activation and priming through a two-step protein cleavage by the transmembrane serine protease 2 (TMPRSS2) [5]. Subsequently, the viral SS+ RNA enters the cytoplasm and undergoes replication, resulting in the production of viral components and synthesis of multiple virions, which are released by exocytosis. The aforementioned steps constitute the basic mechanisms of SARS-CoV-2 life cycle, while repeated cycles finally lead to propagation of the viral infection.

As a result of the propagated infection, there is activation of the immune response and inflammation that begins at the nasopharyngeal mucosa and, if not cleared, continues to the lower respiratory tract. In contrast to 80% of infected individuals who clear the infection early on and have limited inflammation, the majority of the remaining 20% of cases develop a highly inflammatory state, which may eventually lead to Acute Respiratory Distress Syndrome (ARDS) [3]. In the latter group, virions infect the ciliated epithelium of the conducting airways and move downwards to the lower respiratory system. There, they bind to ACE-II enzyme-receptors expressed by type II pneumonocytes and continue a vicious cycle of viral replication and infection of healthy pneumonocytes in the vicinity. As a result, a plethora of pneumonocytes, both type I and type II, are led to apoptosis. The driver of this major inflammatory reaction is the release of a wide variety and amount of pro-inflammatory cytokines from local cells, which lead to a phenomenon called “cytokine storm” [4,6]. These cytokines attract neutrophils, T cells and B cells. The sequestration of inflammatory cells and their attempt for viral clearance cause further damage to the lung tissue and possibly ARDS.

Apart from COVID-19, this type of immunologic cross-talk is also prevalent in cancer, a disease of major mortality in the world today. The multiple-level interaction between cancer cells and the immune system includes early cancer immunoediting, where the tumor immunogenicity is shaped during carcinogenesis, and later cross-talks within the tumor microenvironment, where the most fundamental interactions that will determine cancer cell survival or elimination may occur. Although COVID-19 and cancer sound as diseases of distinct backgrounds, their pathophysiologic mechanisms rely partially on common immunomodulatory reactions driven by the virus or the cancer cells that can finally lead to both positive and deleterious effects on diseases’ progression.

The potential importance and the unclear outcome of an orchestrated immune response against a developing tumor, under a SARS-CoV-2-driven chronic inflammatory status, should be definitely stressed out, in the context of cancer immunosurveillance and the definition of immunoediting and inflammation, as independent or interdependent processes. On this basis, we discuss here whether and to what extent the long-term impacts of COVID-19-associated immunomodulation may interfere with cancer immunosurveillance mechanisms at multiple levels, as well as outline possible immunological interactions underlying a joint pathophysiology of the two diseases that may be worth further investigation.

## 2. Immune Response against SARS-CoV-2

### 2.1. Innate Anti-COVID-19 Response

SARS-CoV-2 stimulates the innate response via introducing pathogen associating molecular patterns (PAMPs) and causing the release of damage-associated molecular patterns (DAMPs). These molecules bind to pattern recognition receptors (PRRs) on innate immune cells leading to increased production of IFN-α and IFN-γ. As such, IFNs establish an inflammatory yet anti-viral state at the early phases of infection [7,8]. However, in a subset of patients, it is likely that instead of the early IFN release, there might be a surge of inflammatory cytokine production, including IL-6 and TNF-α. This is usually attributed to high viral loads or undetermined immunogenetic factors. The cycle of cytokine surge starts with an increase in MCP-1, CXCL-1, CXCL-2, CXCL-5, CXCL-8 and CXCL-10 levels, as a primary response to SARS-CoV-2 infection. The C-X-C motif ligands (CXCLs) then attract and stimulate macrophages and neutrophils in the area of infection [9,10]. As a result, additional cytokines are released such as IL-1β, IL-6 and TNF-α, which in turn further promotes VEGF, IL-6 and IL-8 production, via a feedback loop. The above is the culprit of a phenomenon called “cytokine storm” [11,12]. Moreover, the stimulated neutrophils may produce reactive oxygen species (ROS) that cause further destruction to the site of infection [13], thus suggesting that high neutrophilia in COVID-19 patients may be an independent predictor of poor outcome [14]. Overall, patients with severe COVID-19 infection present with severe lung damage, i.e., ARDS, and/or multiorgan injury [15,16], while they exhibit elevated levels of various cytokines including IL-1β, IL-2, IL-6, IL-8, IL-17, TNF-α, G-CSF, GM-CSF and MIP-1α [3,17,18,19].

Another major component of the innate branch of the immune response against SARS-CoV-2 is the activation of natural killer (NK) cells. NK cells play a crucial role in the first-line defense against viral infections, by inducing cytotoxicity and cytolysis of infected cells [19,20]. Nevertheless, under the prism of the previously analyzed “cytokine storm”, there is an impaired NK representation and function in severely affected COVID-19 patients [21,22]. IL-6 and TNF-α, as the major components of the “cytokine storm’, cause profound depletion and exhaustion of the NK cells [23]. This exhaustion has been mainly attributed to the observed increase in NKG2A expression, an NK receptor known as an important immune checkpoint in NK and CD8+ cells that trims down their cytotoxic activities [21]. Besides the aforementioned components, less important in the innate immunopathology of SARS-CoV-2 infection, is the involvement of complement and the naturally occurring antibodies [18]; thus, they are not further discussed here.

### 2.2. Adaptive Anti-COVID-19 Response

The adaptive immune response against SARS-CoV-2 is highly complicated and not yet fully elucidated. As in most viral infections, the activation of cellular immunity is dominant in SARS-CoV-2-specific recognition and clearance. However, less critical anti-viral humoral responses are also present in severe COVID-19 infections with the production of specific antibodies by hyperactive B cells [24,25], whose amount is correlated with disease severity and the initial viral load [26].

CD8+ T cells are considered the major mediators of adaptive immune responses, as they target and kill virally infected cells. However, in many COVID-19 patients, including the first reported SARS-CoV-2-positive case, have been observed markedly reduced numbers of circulating cytotoxic T cells, B cells and NK cells [27,28]. This lymphopenia is mainly attributed to the fact that SARS-CoV-2 directly infects and causes lymphocytic death and CD8+ T cell exhaustion, via increased programmed cell death protein 1 (PD-1) expression [29,30]. In addition, the sustained lymphopenia caused by severe COVID-19 infection may coincide with an increase in circulating neutrophils, as shown in a COVID-19 patient series [31,32,33]. On the other hand, CD4+ T cells activate CD8+ T cells and B cells and secrete chemotactic cytokines. Notably, memory T helper (Th) cells against SARS-CoV-2 have been detected in uninfected individuals, which have probably been derived by previous exposures to other coronaviruses of the common cold [34]. In addition, in severely infected COVID-19 individuals there has been reported increased numbers of CCR6 CD4+ T cells, thus indicating a potential role of Th17 cells in the immunopathology of the disease [27,35]. Th17 cells produce IL-17, which in turn induce macrophages and dendritic cells [36] to secrete cytokines, thus participating in the cytokine storm.

Overall, of what we know so far, it appears that infection by the SARS-CoV-2 virus causes a dysregulated hyperinflammatory stage, which in turn evolves into an adaptive immunosuppression phase, characterized by peripheral lymphopenia [37,38].

### 2.3. Long-Term Impacts of COVID-19-Mediated Immunomodulation

Quite often SARS-CoV-2 positive status may persist long after the primary infection. The induction of some level of constitutive immune system activation leads to a syndrome called long-COVID syndrome. In addition to the aforementioned lymphopenia, which characterizes the late adaptive response against severe SARS-CoV-2 infection, it might be possible that the reported virus-mediated T cell dysfunction is associated, to some extent, with similar T cell disorganization observed in various autoimmune diseases [39]. This notion is supported by evidence showing an autoimmune-like inflammation in the thyroid gland of some post-COVID patients. There are also reports of increased and persisting auto-antibodies against cells of the innate immune system and anti-viral cytokines, such as IFNs. To reinforce even further the notion of constitutive post-COVID immune system dysregulation, a manifestation of delayed SARS-CoV-2-associated immunomodulation was introduced by the name “multi-system inflammatory syndrome” (MIS). MIS is characterized by high inflammatory status and increased levels of pro-inflammatory cytokines, including IL-6, which are also found in the acute phase of COVID-19 infection. The hyper-inflammatory state can be further promoted by the characteristic lymphopenia and especially the lack of T regulatory cells (Tregs), known to be essential for the regulation and eventual resolution of the inflammation [40]. Overall, comparison of the lymphocyte numbers, types and functions between long-COVID patients (post-acute sequelae of SARS-CoV-2 infection (PASC)) and non-PASC patients revealed declined functions and reduced numbers of IFNγ−/CD107a+ and IFNγ+ CD8+ cells in PASC patients [41].

On top of the long-COVID, a recent hypothesis supporting that multiple possible reinfections may cause a prolonged “on-off” acute inflammatory states, begin to gain ground. Although the chance of symptomatic reinfection has been currently estimated in 0.37% for low-risk patients and in 1.59% for high-risk patients, these percentages tend to elevate over time, as new and more complex variants of the virus emerge [42]. In addition, while many scientists agree on the existence of the long-COVID syndrome, an ongoing debate regarding the incidence, duration, and symptomology of “long-COVID” has recently initiated. According to a recent large retrospective cohort, more than 60 non-specific symptoms were associated with SARS-CoV-2 infection after 12 weeks post-detection, thus suggesting that this syndrome may be presented with a plethora of putative phenotypes associated with a range of sociodemographic and clinical risk factors [43]. Therefore, it becomes apparent that a considerable amount of time and relevant studies are required for better understanding of the syndrome and its specific symptomatology, especially when issues regarding its relationship with other medical entities are raised by the scientific community.

## 3. Cancer Immunosurveillance, Immunoediting and Inflammation

### 3.1. The Basic Concepts of Cancer Immune-Surveillance and Immune-Escape

Following the “cancer immune-surveillance theory”, addressed by Thomas and Burnet in the early 1950s [44,45], Shreider and colleagues came up in 2002 with a more comprehensive theory that first introduced the term “cancer immunomodulation or immunoediting” in an effort to explain the different immunogenicity of cancer cells and the underlying mechanism(s) that causes it [46]. According to this theory, known as “cancer immunoediting theory”, the relationship between immune system and cancer cells follows an evolutionary course of three phases, which ultimately leads to carcinogenesis and the selection of more aggressive and immunoresistant cancer phenotypes as the disease progresses. The first phase, known as “elimination”, represents the period of effective immunosurveillance, where the immune system efficiently recognizes and eliminates mutated and cancer cells, thus preventing tumor establishment and growth. In the second phase, known as “editing”, those cancer cells that have gradually developed the ability to bypass the immune monitoring mechanisms begin to prevail. Therefore, these cells form an immunogenic phenotype shaped by the evolutionary drive that the host’s immune mechanisms impose. From this point on, the balance between efficient immunosurveillance and oncogenesis closes towards oncogenesis. In the third phase, known as “escape”, cancer immunosurveillance is no longer able to control the proliferation and spread of immunoresistant cancer cell phenotypes. As a result, these phenotypes completely dominate and advance towards clinically significant tumors.

Therefore, the core of the “three Es” theory is that the immune system, while it protects the body from cancer cell prevalence and spread, also forms the immunogenic identity of tumors (immunomodulation). In addition, it makes clear that the mechanisms of tumor escape from host’s immunosurveillance represent the end-result of a long-lasting interaction of the mutated/cancer cells with the immune system, during the second phase of cancer immunoediting. As such, the formation of the immunoresistant cancer cell phenotypes may be the result of the evolutionary pressure that the immune system exerts on all mutated/cancer cells, along with the high tumor heterogeneity and genomic instability that further force selection and survival of the most resistant cells. Thus, while at first glance the communication between the immune system and cancer cells appears one sided, new evidence suggests an interplay between the two system components with cancer cells having the ability to epigenetically modify immune cells, which ultimately contributes to the reduction of their activity as tumor suppressors [47,48].

Tumor immune-evasion can be mediated by multiple mechanisms that elude cancer cell destruction by innate and adaptive immunity, while they involve both cancer and immune cells [49]. The central role, in the aforementioned process, is played by a subpopulation of malignant cells known as cancer stem cells (CSCs), which usually present with complex immunoresistant properties [50]. CSCs have the unique ability of self-renewal, while they can easily give distant metastases after they undergo epithelial to mesenchymal transition (EMT) [51]. Oncogenic EMT is usually associated with changes in hallmark transcription factors, including Snail [52], which has been associated with reduced activity of T-cell mediated cytotoxicity, while it promotes further immunosuppression by forcing the production of suppressive cytokines such as IL-10 and TGF-β.

Moreover, among the most prevalent mechanisms, associated with tumor escape from host adaptive immunosurveillance, are thought to be expression changes in significant membrane proteins on immune and cancer cells. These changes include upregulation of the inhibitory immune checkpoint receptors PD-1 and CTLA-4 [53,54] and downregulation of co-stimulatory molecules (e.g., CD28) on T cells, as well as reduction of the major histocompatibility complex (MHC) antigens class I, increase of the inhibitory ligand PD-L1 [53] and reduced presentation of tumor specific (TS), or tumor associated (TA) antigens on cancer cells [49,55]. The MHC molecules are subject to down-regulation on cancer cell membranes, via gene mutations, gene deletions and epigenetic alterations [50,56]. PD-L1 binding to PD-1 receptor on T cells causes CD8+ cytotoxic T cell (CTL) exhaustion and dysfunction [53]. Along with CTL exhaustion, death or reduced infiltration into the tumor microenvironment, CD4+ T cells in resistant tumors tend to polarize into the T helper 2 (Th2) and regulatory T (Treg) cell phenotypes. Tregs and myeloid derived suppressor cells (MDSCs) are major suppressors of the CTL-mediated cytotoxicity against cancer cells, while their high incidence within the tumor microenvironment is considered a marker of poor prognosis [57,58].

Failure of adaptive immunity activation in cancer may be also initiated by poor tumor antigen presentation by dendritic cells which lack efficient maturation as well as by the polarization of macrophages into the immunosuppressive M2 phenotype [59]. Malignant cells may further poorly respond to attraction, recognition and cytotoxic and phagocytotic activities of innate immune-subpopulations, including macrophages, neutrophils and NK cells, via downregulating chemokine receptors and stress ligands or by producing negative signals for efficient recognition. Last but not least, changes in the prevalence of highly immunosuppressive soluble factors produced by cancer and immune cells, within the tumor microenvironment, may further contribute to tumor immune-evasion [47,49].

### 3.2. Cancer Immunosurveillance vs. Inflammation: The Inflamed Tumor Microenviron Ment

Inflammation constitutes the “yin/yang” driving force for either resolution or maintenance of a variety of disorders, including, among others, infectious diseases and cancer. Inflammation can be divided into its acute and chronic forms, from which the chronic form has been mostly associated with oncogenesis and cancer progression [60]. Nevertheless, the implication of acute inflammation within the tumor microenvironment (TME) in cancer advancement has been also hypothesized, as both acute and chronic inflammations share some common effectors. This hypothesis was tested in murine models of breast cancer, where a significant increase in lung metastasis was observed immediately after induction of traumatic/acute topical inflammation. The findings were attributed to immune cell accumulation and IL-6 overproduction in the lung, thus supporting the possible involvement of acute inflammation in disease progression [61,62].

Chronic inflammation has been considered a crucial culprit behind the creation of a pro-tumorigenic microenvironment, which further supports cancer advancement [60]. Along with the infiltration of the various immune cell subtypes, the constitutive production of specific cytokines, including IL-6, IL-10, TNF-α and TGF-β, sustained by the chronic inflammatory background, advances cell communications and changes that may contribute to cancer development and eventually to disease progression. Specifically, IL-6 constitutes a major promoter of tumor growth by activating the JAK/STAT3 pathway [63] and by inducing oncogenic epithelial to mesenchymal transition (EMT). TNF-α/IL-6 synergism has been reported to slightly advance TGF-β-mediated EMT [64,65], while IL-6 by itself can upregulate EMT markers like vimentin, whereas reducing the expression of E-cadherin via the JAK/STAT/Snail pathway, thus increasing the invasive potential of the tumor [66]. Furthermore, IL-6 promotes angiogenesis through induction of VEGF [67,68].

TME is also enriched in IL-10, a multifunctional cytokine, secreted by nearly all leukocytes. IL-10 exerts immune cell-type specific distinct effects with the anti-inflammatory and immunosuppressive functions to be the most dominant [69]. Whereas IL-10 produced by T cells is required to control chronic inflammation, it seems to be dispensable during acute inflammation [70]. In the context of the anti-inflammatory response, IL-10 binds to its cognate receptor IL-10R, leading to activation of the IL-10/Jak1/STAT3 cascade [71]. The phosphorylated STAT3 in turn promotes the transcription of target genes, among which are the anti-inflammatory response (AIR) factors, thus resulting in the suppression of the pro-inflammatory gene expression [72]. Thus, while one may expect that increased IL-10 levels within the TME would reduce the tumorigenic inflammation, there appears to prevail a status of sustained hyperinflammation, along with a simultaneous immunosuppression attributed to STAT3 activation [73]. IL-10-mediated STAT3 phosphorylation is known to enhance the differentiation of immature Tregs towards an immunosuppressive phenotype [74], while it has potent anti-apoptotic effects through upregulation of Bcl-2 [75,76]. Additionally, IL-10 accumulation within the TME reduces the functions of DCs as it decreases their ability to secrete IL-12, thus inhibiting the function of the cytotoxic T cells, and leading to local immunosuppression and activation of factors causing resistance to chemotherapy [77]. On the other hand, TNF-α, an inflammatory mediator known to participate in the pathophysiology of chronic inflammatory diseases, is also highly present in TME. TNF-α, among other functions, has been implicated in the early stages of carcinogenesis as it promotes ROS and RNS formation, which cause DNA damage and mutations [78,79]. Finally, TGF-β, a primarily immunosuppressive cytokine, mediates TME immunosuppression by inducing cytotoxic T cell immunotolerance and decreasing the effectiveness of NK cell-related cytotoxicity [80,81]. More prominently, TGF-β is also highly correlated with oncogenic EMT promotion via complex interactions leading to the activation of NF-κB and JAK/STAT pathways as well as with acquisition of cancer stem cell phenotypes [82,83].

Noteworthy, apart from the critical contribution of the immune cell products like the cytokines described above in establishing a chronically inflammatory pro-tumorigenic or tumor advancing microenvironment, the immune system can contrastingly play a pivotal role in cancer immunosurveillance and immunoediting. Interestingly, recent evidence suggests that these oppose functions of the immune system can coexist, even in the same tumor microenvironment, as many of its components may function differently, depending on the circumstances. Therefore, there is no dispute on the immune system bipolarity regarding its pro-tumorigenic or anti-tumorigenic effects [84,85].

Examples of the bifunctionality of the immune cell populations and products in cancer development and progression are worth mentioned. TGF-β displays contrasting roles in inhibiting tumor initiation while promoting tumor invasion at late stages [86]. Similarly, mice lacking TNF-α, a well-known pro-tumorigenic effector, were shown to be more susceptible in forming chemically induced sarcoma [87]. TNF-α may also coexist with the apoptosis inducer TNF-related apoptosis-inducing ligand (TRAIL) in the same TME. In this case, the dominant signal is dependent on the NF-κB activation status, as active NF-κB favors TNF-α expression, whereas its absence promotes TRAIL production [88]. The bifunctionality of the immune system on cancer onset and advancement further expands onto the immune cells as well. For example, while IFN-γ-producing NK cells have direct cytotoxic effects on cancer cells [89], they can simultaneously suppress the anti-tumor functions of dendritic cells [90]. Moreover, the Treg-mediated immunosuppression can both promote cancer [91] and inhibit the cancer promoting chronic inflammation [92]. Even CD8+ T cells may have dual functions within the TME, as they produce and are affected by tumor-promoting cytokines [93], while they are the primary and direct cytotoxic effectors on cancer cells [94].

Overall, almost every cytokine and immune cell population existing within the pro-tumorigenic or established tumor microenvironment may have a putative dual functional role in oncogenesis and tumor progression. It is important that further research is needed to shed more light on the exact mechanisms of each effector by customizing its approach to the specific scenario at hand. The inflamed microenvironment is interestingly a place where the tumor-promoting chronic inflammation and cancer immunosurveillance probably coexist, even though they have opposite effects. Although it is shown that chronic inflammation is basically a tumor promoter, the immunomodulation may also have anti-tumor effects. Thus, it is generally accepted that the immune system–cancer interaction should be studied as a “scale”, which can tilt towards different directions, depending on the background processes and stressors.

## 4. COVID-19 and Cancer

### 4.1. COVID-19 Impact on Cancer Disease: The Current Status of Knowledge

High risk subpopulations, such as cancer patients, are more susceptible to SARS-CoV-2 infection and COVID-19 severity, as they are considered immunocompromised due to the malignancy itself and the immunosuppressive therapies they receive, as part of their therapeutic plans [95]. COVID-19 early symptoms and changes in inflammatory markers in cancer patients are mainly non-specific and comparable to patients without cancer [96]. However, regarding the clinical complications, apart from the bilateral lung insult and respiratory symptoms, SARS-CoV-2-infected cancer patients tend to present with serious complications, such as ARDS and embolic phenomena [97]. In addition, further meta-analyses showed worse outcomes for these patients, associated with prolonged hospitalization and ICU stays, as well as with higher risk of mortality [96]. Interestingly, tumor stage and aggressiveness seem to promote COVID-19 severity, as infected patients with metastatic solid tumors and hematologic malignancies tend to have increased mechanical ventilation needs and mortality rates [98]. It is also shown that mortality in the hospital setting for COVID-19-infected cancer patients is five times greater than the mortality of COVID-19 non-elderly and predisposing-condition-free patients [96,99]. However, in a retrospective cohort [100], the adverse outcomes were mainly attributed to the comorbidities that cancer patients present with, such as obesity, active smoking and old age. Moreover, although preliminary findings did not manage to support a significant association between recent cytotoxic chemotherapy treatment and adverse COVID-19 outcomes [101], later studies using larger cohorts demonstrated that cancer patients receiving chemotherapy or chemoradiotherapy had also an increased risk of worse outcomes, especially when the anticancer treatment had been administrated shortly before infection [98]. On the flip side, no great difference was observed between the general population and cancer patients undergoing radiotherapy regarding the risk of developing serious COVID-19 illness, since cancer patients may have increased immune activity derived from the radiotherapeutic effects on the immune system [102,103]. Importantly, concerning the checkpoint inhibitor immunotherapies, there has been difficulty in discerning the immune related toxicities of these treatments from the clinical manifestations of COVID-19 [104].

COVID-19 severity and cancer cross-talk has been approached in the literature in a bidirectional basis. As such, severe infection with SARS-CoV-2 may interfere with the malignant status and promote cancer progression, at the molecular and cellular levels via multiple signaling pathways [105]. For example, high levels of angiopoetin-2, a molecule which increases angiogenesis, is linked with severe ARDS, while it is capable of promoting new vessel formation and subsequent cancer progression in cancer patients with severe COVID-19 illness [106]. Moreover, heat shock protein 27 (HSP-27) phosphorylation has been positively associated with cancer aggressiveness. COVID-19 induces downregulation of ACE-II leading to increased bradykinin which in turn activates the mitogen activated protein kinase (MAPK), a kinase responsible for HSP-27 phosphorylation, thus contributing to cancer progression [107,108,109]. In addition, COVID-19-mediated ACE-II downregulation shifts angiotensin I (AT-I) into the angiotensin converting enzyme/angiotensin II/angiotensin II type I receptor pathway (ACE/AT-II/AT-1R), thus increasing the activity of angiotensin II (AT-II). This shift leads to a state of increased inflammation and oxidative stress [110]. Findings from in vitro studies have suggested that AT-II is implicated in cancer stem cell emergence in small cell lung carcinoma and angiogenesis in breast cancer [111,112]. Lastly, recent evidence supports that there is involvement of TMPRSS2 both in COVID-19 and prostate cancer molecular pathophysiology, through its association with the ACE-II and androgen receptors, respectively [113].

However, while the majority of the literature is in support of this positive correlation between COVID-19 severity and cancer progression, a significant number of recent case reports about cancer remission during or after SARS-CoV-2 infection cannot remain undisputed. These cases concern a variety of cancer types including colorectal cancer, cutaneous T-cell lymphoma, EBV positive Hodgkin lymphoma, NK/T-cell lymphoma, follicular lymphoma, multiple myeloma and acute leukemia [114,115,116,117,118,119,120]. Direct oncolysis, immune system activation and epitope spreading are among the prevailing underlying mechanisms currently proposed for the SARS-CoV-2-mediated cancer suppressive effects [121]. However, questions arise about whether these effects can be attributed to SARS-CoV-2 infection itself, or they are a consequence of a possible interaction between the COVID-19 treatment modalities and the cancer cells, or the anti-cancer therapies. Notably, the cases of COVID-19-associated cancer remission have raised important discussion within the scientific community, regarding the potential use of SARS-CoV-2 as an oncolytic virus for cancer virotherapy [122]. However, recent findings are discouraging on SARS-CoV-2 candidacy, as it does not show oncotropism and infects equally efficiently normal cells [123]. Furthermore, this virus has high rate of mutations, thus making it unstable and potentially harmful to cancer patients [124].

### 4.2. Immunologic Interplay between COVID-19 and Cancer Onset/Progression

Although cancer immunosurveillance and tumor-promoting inflammation can co-exist in the same microenvironment as mentioned above, the condition that will finally prevail depends on the circumstances that will turn out to be favorable for one condition or the other. The exact mechanisms, by which cancer immunosurveillance and the pro-tumorigenic microenvironment are affected by the inflammatory stress introduced by this relative new virus, are not yet clearly elucidated. The whole spectrum of chronic and cancer related effects of SARS-CoV-2 need more time to be fully unraveled because “cancer immunoediting” is a process that requires a considerable amount of time to develop. Based on the current understanding of the pathophysiology of COVID-19 and cancer onset and progression, the following putative scenarios of immunologic interplay between the two diseases can be suggested.

**Figure 1:**
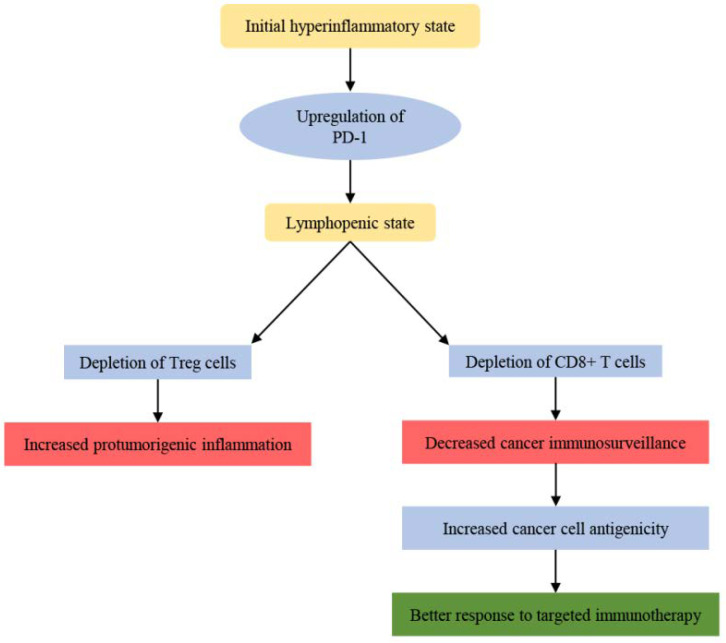
The putative dual effect of COVID-19 induced lymphopenia on cancer. The initial hyperinflammatory state caused by COVID-19 is followed by a lymphopenic state that affects both Tregs and CD8+ T cells with different end results in cancer growth and treatability.

The hyperinflammatory state, caused by severe SARS-CoV-2 infection, is followed by a state characterized by lymphopenia. This lymphopenia can be attributed to the upregulation of immune checkpoints on immune cells, like PD-1 [30], caused by direct SARS-CoV-2 infection of the immune cells. At a first glance, this can be interpreted as a tumor promoting effect, as there is induction of pro-tumorigenic inflammation with concurrent inhibition of cancer immunosurveillance. The pro-tumorigenic inflammation may be in part attributed to the fact that Tregs, which reduce the inflammation, are depleted, whereas the inhibition of cancer immunosurveillance can arise by possible loss of CD8+ T cells. Based on the above scenarios, one can easily hypothesize that there will eventually be a potential increase in cancer aggressiveness. However, there is also a possibility that the cancer cells, which develop in this lymphopenic environment, have greater antigenicity [84], and therefore they can be more easily targeted by corresponding therapies. This greater antigenicity is proposed on the basis of the immunoediting theory, according to which, cancer cells that do not have constant and strong interaction with the immune system, most likely will not have the drive to evolve into immunoresistant cell phenotypes [44,45] (Figure 1).

NK cells may further be catalytic players in COVID-19/cancer immune-surveillance crosstalk. Normally, NK cells recognize and eliminate, through apoptosis, cells that have down-regulated MHC I antigens, like the highly mutated and the already malignant cells [125]. In an environment of COVID-19-induced “cytokine storm”, NK cells are exhausted and depleted, thus eliminating their anti-cancer cytotoxic activities [21]. On the flip side, this exhaustion may have a disinhibitory effect on the NK-mediated suppression of dendritic cell functions [90], thus allowing the latter cells to exert their anti-tumor activities.

Apart from the immune cell types, the cytokines that constitute main components of the COVID-19-mediated “cytokine storm” may also be considered as putative drivers of an immunologic interplay between the two diseases. IL-6 is evidently leading the development and effects of “cytokine storm” in severe COVID-19 [12], while it has a major tumor-promoting role as it activates the IL-6/JAK/STAT pathway and induces the invasive and angiogenic properties of cancer via interactions with other cytokines [63]. As such, IL-6 may be a putative interlink of COVID-19/cancer interplay; however, it remains unclear whether the duration of the “cytokine storm”, presently known as an intense yet acute inflammatory phenomenon, would let enough time for IL-6 to exert its tumor-promoting effects.

An immunologic interlink could be further supported by the fluctuating expression levels and the relevant effects of additional cytokines that also participate in the “cytokine storm” phenomenon, including TNF-α increase and IFN type I drop. Although, TNF-a is basically a pro-tumorigenic cytokine, it shows, like many other immunologic effectors in the tumor microenvironment, a dual functionality, which makes it difficult to puzzle out its true effects on cancer immunosurveillance [88]. On the flip side, it is well documented that IFN-I inhibition favors cancer immune evasion [126], while its pathologically reduced levels have been associated with severe COVID-19 [10]. Therefore, the putative effects of COVID-19-mediated IFN-I downregulation are speculated to induce cancer immune escape, however this hypothesis remains to be validated. 

Another interesting question one can address is what would be the possible effects of the complex phenomenon of long-COVID on cancer immunosurveillance. As mentioned above, the long-COVID syndrome is the chronic manifestation of COVID-19 and is characterized by a low potency chronic inflammatory status that affects multiple organ systems [40,127]. The chronic inflammation has been well-characterized as a tumor promoting background, which along with the reported COVID-19-associated lymphopenia may further tilt the balance towards tumor promotion. On the other hand, T cell abnormalities frequently observed in long-COVID are comparable to those in autoimmune diseases [39]. This suggests that their substantial hyperactivity and dysregulation may tilt the balance to both sides. However, the ambiguity surrounding the definition of this medical entity is an additional barrier against its association, positive or negative, with oncogenesis and/or cancer progression [43]. Therefore, it is important to evaluate the quality of this inflammation since different quantity of various effectors may drive the balance between cancer immunosurveillance and tumor promoting inflammation towards totally opposite directions.

On the basis of the aforementioned comparable immune dysregulation caused by autoimmunity and COVID-19, and in support of our hypothesis for an immunologic interplay between COVID-19 and cancer onset and progression, there are well documented associations among multiple autoimmune diseases and certain cancer types, e.g., systemic lupus erythematosus (SLE) and diffuse large B cell non-Hodgkin lymphoma [128], as well as rheumatoid arthritis (RA) and malignancies of the hematopoietic lineage [129]. Substantial evidence further demonstrates IL-6 as a common player in autoimmunity- cancer crosstalk. For example, severe RA symptoms are tightly correlated with elevated IL-6 levels in the synovial fluid [130], while IL-6 overproduction has been associated with poorer outcomes of multiple myeloma [131] as it may promote the growth of myelomas and plasmacytomas [131,132]. In addition, IL-6-producing cardiac myxoma presents with autoimmune-like symptomatology [133], while prostatic intraepithelial neoplasia (PIN) shows increased IL-6 expression [134]. These findings suggest that IL-6 may trigger and link processes including chronic inflammation, autoimmunity and various malignancies [135].

Other studies further highlight connecting mechanisms of immune-related adverse events derived by cancer immunotherapy with PD-L1 immune checkpoint inhibitors and autoimmunity [136]. In this context, it has been proposed that the immune dysregulation caused by immunotherapies and autoimmune diseases may contribute to increased viral associated cancers since the clearance of oncogenic viruses may be impeded [137,138]. Examples of viruses that thrive in this kind of environment are HPV, EBV and HBV/HCV. Notably, it is shown that a heavy infectious blow to the immune system caused by the immunomodulatory virus HIV, which primarily infects immune cells, can be the culprit of multiple malignancies that appear frequently in AIDS patients [139]. As such, many HIV-associated malignancies have been mainly attributed to insufficient clearance of certain oncoviruses including HHV-8, EBV and HPV, which can trigger Kaposi sarcoma, lymphomas and gynecological cancers, respectively [140]. Interestingly, the use of highly active antiretroviral therapy (HAART) appears to lower the incidence of HIV-associated cancers in HIV-infected patients [141,142].

Likewise, SARS-CoV-2 is a virus that strongly interacts with the immune system and the results of this relationship, which we are observing nowadays, may only be the tip of the iceberg. Therefore, it is of crucial importance to illuminate all the immunomodulatory aspects of this high-prevalent virus in order to comprehend its true effects on cancer immunosurveillance mechanisms.

## 5. Conclusions

The study of COVID-19 epidemiology and pathophysiology has remained in the center of the scientific attention since early 2020. The growing new bio-information on the topic has sparked intense debate on various aspects of virus–host interactions and important questions remain to be answered. Infection by the SARS-CoV-2 virus is causing a series of immune-mediated and immune-modulated responses, which have not been fully elucidated yet. In the context of the immunological approach of the onset and progression of many diseases, including cancer, there is substantial evidence suggesting that the durability and severity of COVID-19-associated inflammatory status may interfere with the effectiveness of the immune-monitoring mechanisms during cancer establishment and advancement.

Although it is still too early for safe predictions and discriminations of the magnitude of the long-COVID-19 impact on the appearance and pathophysiology of solid and hematological tumors, based on the discussion on IL-6-mediated effects on B cell malignancies [131,132], one could speculate that the incidence and progression of hematological malignancies are more likely to be directly and more intensively affected by serum levels of certain cytokines that are present in the virus-induced cytokine storm and have constantly remained increased during long-COVID syndrome. This notion is further supported by examples of other immunomodulatory viruses, like HIV, that primarily affect the immune system and increase the risk for lymphomas [140]. However, it seems that several solid malignancies are also affected, mainly towards progression [140]. In addition, it still remains unclear how immunologically protected cancers, like those of the central nervous system and the testicles, respond to COVID-19-mediated immunomodulation, even though reports on the ability of SARS-CoV-2 to dysregulate both the blood–brain and the blood–testes barrier and infect those tissues are available [143,144].

Overall, the associated evidence and hypothetic scenarios on a putative COVID-19/cancer interplay described above may be worth of further investigation, so the exact mechanisms and joint pathophysiology of the two diseases may be fully elucidated, if they indeed exist. However, the timeline of these investigations, especially those related to the impact of COVID-19 on cancer incidence, is also difficult to be safely predicted, given that a respectable amount of time is required for accumulation of all the necessary genetic alterations that will transform a normal cell into a malignant one. In addition, even after the first malignant cell emerges, there is a huge latency period until this cancerous cell evolves into a malignant tumor that can be diagnosed, due to the time consuming three-stage cancer immunosurveillance process [46,84]. On top of these, the vaccines and the anti-viral medications against SARS-CoV-2 are capable to boost the immune response against the virus and potentially weaken the putative link between long-COVID syndrome and cancer incidence and progression.

Summarizing, it is understandable that if the de novo COVID-19-induced oncogenesis exists, it is most likely a multi-parametric equation. Thus, our speculations on the ideal timeline of relevant investigation studies are highly hypothetical. However, we strongly believe that research proposals aiming to examine possible induction of hematological malignancies by SARS-CoV-2 infection worth immediate initiation, as the minimum latency time is approximately 140 days for lymphoproliferative and hematopoietic cancers, whereas approximately 4 years for solid malignancies [145]. Additionally, we suggest reinforcement of the current research efforts restricted to COVID-19-induced cancer progression in already diagnosed malignancies, as the investigation parameters can be measured and immediately compared with previous measurements in a less time-consuming way, thus conferring to easier planning and execution of the research projects.
